# Time of day influences memory formation and dCREB2 proteins in *Drosophila*

**DOI:** 10.3389/fnsys.2014.00043

**Published:** 2014-03-31

**Authors:** Robin Fropf, Jiabin Zhang, Anne K. Tanenhaus, Whitney J. Fropf, Ellen Siefkes, Jerry C. P. Yin

**Affiliations:** ^1^Departments of Genetics, University of Wisconsin-MadisonMadison, WI, USA; ^2^Neuroscience Training Program, University of Wisconsin-MadisonMadison, WI, USA; ^3^Department of Neurology, University of Wisconsin-MadisonMadison, WI, USA

**Keywords:** time-of-day, memory formation, dCREB2, sleep, transcription, genetic

## Abstract

Many biological phenomena oscillate under the control of the circadian system, exhibiting peaks and troughs of activity across the day/night cycle. In most animal models, memory formation also exhibits this property, but the underlying neuronal and molecular mechanisms remain unclear. The dCREB2 transcription factor shows circadian regulated oscillations in its activity, and has been shown to be important for both circadian biology and memory formation. We show that the time-of-day (TOD) of behavioral training affects *Drosophila* memory formation. dCREB2 exhibits complex changes in protein levels across the daytime and nighttime, and these changes in protein abundance are likely to contribute to oscillations in dCREB2 activity and TOD effects on memory formation.

## Introduction

The daily oscillation between light and dark periods is one of the most significant changes that all animals experience. These circadian states heavily influence fundamental behaviors such as feeding, mating, and sleeping. As such, it is intuitively appealing that this system has large effects on cognitive behavior. This instinct likely results from the circadian control of sleep and the universal human experience that grogginess is not usually conducive to cognitive processes. But in most experimental contexts, this modulatory state is controlled for and does not contribute to the observed behavioral data (Gerstner et al., 2009). Circadian effects on cognition involve at least two separable processes. First, animals from *Aplysia* to humans show variable learning and memory formation capabilities across the day/night cycle (Gerstner et al., 2009). In the related observation, time stamping, animals trained at a particular time during the circadian cycle invariably perform the best when subsequently tested at that time (Holloway and Wansley, 1973). In this report, we will only discuss the variable effects of training time. However the systems, cellular, and molecular mechanisms that contribute to either aspect of this phenomenon are just beginning to emerge.

Elegant experiments using hamsters show the relationship between the circadian system and memory formation. Specific manipulation of the light/dark cycle results in animals that are behaviorally arrhythmic, but free from the pleiotropic effects that plague genetic disruptions or the anatomical confounds of tissue ablation (Ruby et al., 2008, 2013). These arrhythmic animals no longer display a circadian preference for learning/memory of a novel object recognition task. However, if GABAergic neurotransmission is blocked, the circadian effect returns, suggesting that inhibitory neurotransmission onto the hippocampus gates the performance of this task. This work demonstrates that systems-level influences are involved in circadian effects in the mammalian brain and that these processes could involve inhibition onto the areas where memory-induced changes occur.

At the molecular level, one signaling cascade important for memory formation is the ERK-MAPK pathway. This essential signaling molecule oscillates in its activity state, including in brain regions involved in memory formation (Eckel-Mahan et al., 2008). Further, long term memory expression relies on intact MAPK oscillations. Experiments that block post-training hippocampal MAPK peaks in activity inhibit memory consolidation, while inhibition during troughs in activity have no effect (Eckel-Mahan et al., 2008). These results demonstrate that naturally occurring oscillations in molecular pathways involved in memory formation are likely to be crucial for memory maintenance.

The cAMP/PKA/CREB signaling pathway is a second conserved molecular cassette that is important in both circadian and cognitive processes. In mammals, light entrainment of the circadian clock requires CREB activity (Ding et al., 1997). However, the signaling pathways leading from light presentation to CREB activation may differ depending upon the time of night. A light flash delivered during the early nighttime phase delays the circadian clock, light presentation late at night phase advances the clock, and light exposure during the middle of the night has no effect. The signaling requirements during the early and late night times differ, and one model is that cAMP/PKA serves as a molecular gate for phase shifting, allowing it to occur during the early nighttime, but inhibiting part of the process late at night. Currently, the details of these mechanisms are unclear (Ding et al., 1997, 1998; Tischkau et al., 2000, 2003; Cao et al., 2013).

The cAMP/PKA/CREB pathway constitutes a vital component in the acute changes that occur during memory formation. Manipulating different components of the pathway can produce memory enhancement or inhibition (Kandel, 2012). Recent work suggests that the pathway also has a more chronic, sustained role in fundamental neuronal properties such as intrinsic excitability (Benito and Barco, 2010). Exactly if and how excitability influences overall memory formation remains unclear. An intriguing possibility is that a role in “(re)setting excitability” could be related to a long-term role in the maintenance of memories.

Circadian influences on learning and memory formation have also been reported in invertebrates. Early work in Aplysia showed that the time of training affects sensitization, a non-associative form of behavior, as well as associative behavior (Fernandez et al., 2003). This finding constituted a significant advance, as it reframed the question of circadian effects and moved beyond the instinctive notion that training time influences memory formation primarily through sensory gating. More recently, the circadian system has been shown to influence different behavioral paradigms and various phases of memory in Aplysia (Lyons et al., 2005, 2006; Lyons and Roman, 2008). In *Drosophila*, the time of behavioral training has been shown to affect learning, defined as the immediate acquisition and retention of associative information (Lyons and Roman, 2008). However, it has not been demonstrated in flies that the time of behavioral training can influence longer lasting forms of plasticity, including long-term memory.

The *Drosophila* dCREB2 gene has been shown to be important for circadian rhythms in flies (Belvin et al., 1999). A dCREB2- and CRE-dependent transgenic reporter (CRE-luciferase) shows an oscillatory pattern in activity, with peaks in the middle of the daytime and nighttime periods. Flies that contain a stop codon in the dCREB2 gene (S162) show a shortened circadian periodicity. Moreover, both the oscillatory pattern and the activity of the core clock Period and Timeless proteins are affected in the S162 mutant background. Finally, the oscillatory pattern of the CRE-luciferase reporter is under circadian control, corresponding to the periodicity of the fly. These results led to the conclusion that dCREB2 is part of the circadian clock, probably via transcriptional feedback loops that affect *period* expression. dCREB2 has a well established role in long-term memory formation (Yin et al., 1994, 1995a; Chen et al., 2012; Hirano et al., 2013; Tubon et al., 2013). Since dCREB2 is involved in both processes, it is logical to determine if it serves as a molecular integration point between these two systems.

In this study, we show that the TOD of behavioral training influences long-lasting memory formation. Because of the central role that dCREB2 plays in memory formation and its connections with circadian biology, we analyze dCREB2 protein isoforms across the day/night cycle. Correlations between behavior and dCREB2 protein profiles suggest that it contributes at the molecular level to TOD behavioral effects.

## Materials and methods

### Animals

Fly stocks were maintained at 23°C on standard food. Experimental animals were collected 1–3 days post eclosion, housed at 100 flies per vial, and entrained to a 12:12 light:dark schedule for 3–5 days before behavioral or molecular experiments. Initial time-of-day experiments were performed with Canton S wild-type flies. Further behavioral experiments and expression experiments following behavioral training utilized the *w iso(CJ1*) strain, known colloquially as 2202U.

### Bioluminescence measurements

The *in vivo* luciferase assay was performed as described previously (Brandes et al., 1996; Stanewsky et al., 1997; Belvin et al., 1999; Tanenhaus et al., 2012). Briefly, flies carrying an upstream activating sequence driven flippase and a flippase-dependent CRE-luciferase reporter (UAS-FLP; CRE-F-luc) were crossed to wild type (yellow white) or GAL4 driver flies that carry a transgene to drive targeted expression of the yeast transcriptional activator GAL4 in specific cell types. The progeny were entrained through development to a 12:12 light:dark cycle and loaded individually into 96 well plates containing luciferin-fortified food. Luminescence was measured hourly using a TopCount microplate scintillation and luminescence counter (PerkinElmer). After 2 complete days, the plates were switched to constant darkness. The first 11 h were excluded from analysis to allow for initial substrate feeding. A smoothing function is applied such that each data point represents the average of three measurements.

### Behavioral training

Flies were trained in the olfactory avoidance-training paradigm developed by Tully and Quinn and modified to allow for automated training sessions (described in Fropf et al., 2013). This protocol causes animals to form an association between an electric shock (the unconditioned stimulus) and a previously neutral odor (the conditioned stimulus). Each electric shock consists of 1.5 s 70-V pulses administered every 5 s over the course of 60 s (12 total). A single-cycle of training involves 90 s exposure to ambient air; 60 s of electric shock accompanied by simultaneous exposure to first odor (the conditioned stimulus condition, CS+); 45 s of ambient air exposure to clear the first odor; 60 s of exposure to the second odor, with no shock (the CS− condition), 45 s of ambient air to clear the second odor. Spaced training consists of the specified number of single cycles separated by 15-min rest intervals. 3-octanol and 4-methylcyclohexanol are used as odors.

To test memory or learning, flies are placed in a choice point and allowed to decide between the CS+ and CS− stimuli for 90 s. The performance index = [the number of flies making the correct choice] – [the number of flies making the incorrect choice]/total number of flies, multiplied by 100. To avoid odor-avoidance biases, we calculate the performance index by taking an average performance of two groups of flies, 1 trained with 3-octanol as CS+, the other with 4-methylcyclohexanol. This average of the two groups constitutes an *N* = 1.

### Antibodies

The antibodies used in this study and their epitopes have been previously published (Fropf et al., 2013). The αPan and αPO_4_ antibodies are polyclonal antibodies raised in rabbits. The αPan antibody was raised against a sequence of amino acids in the basic region of dCREB2, and affinity purified using the antigenic peptide linked to a column. The αPO_4_-specific antibody was raised against a peptide that contains the S231 residue in a phosphorylated state. Serum was passed over a peptide column containing the unphosphorylated peptide, and the flow through fraction was bound and eluted from the phospho-peptide column. The αLamin antibody used in this study is a monoclonal antibody raised in mouse, purchased from *Drosophila* Studies Hybridoma Bank (ADL67.10) and used at a 1:1000 dilution. The αHSP70 antibody is a monoclonal antibody raised in mouse purchased from Sigma (Clone BRM22, Catalog # H5147) and used at a 1:30,000 dilution.

### Tissue preparation for western blot

Sample preparation procedures have been previously published (Fropf et al., 2013). Briefly, flies were flash-frozen in liquid nitrogen and heads were isolated using sieves on dry ice. To prepare nuclear and cytoplasmic fractions from collected heads, heads were homogenized on ice 3 times, for 30 s, in a homogenization buffer containing 15 mM Hepes, pH 7.5, 10 mM KCl, 5 mM MgCl_2_, 0.1 mM EDTA, 0.5 mM EGTA, 1 mM dithiothreitol, 1 mM phenylmethylsulfonyl fluoride, and protease inhibitor cocktail tablets (Roche cOmplete ULTRA Tablets, Mini, EDTA-free, EASYpack 05 892 791 001). Debris was eliminated using three 5 min 200 g spins and supernatant was collected each time for the subsequent spin. To collect nuclear sample, pellets were saved and combined from three spins at 1000 g for 5 min. Nuclear pellets were washed once with homogenization buffer and re-spun for purer nuclear samples. Cytoplasmic samples consisted of the supernatant following the third nuclear spin, which contained the carry over, non-nuclear material from the previous two spins. Protein concentrations were determined using a Bradford assay and normalized across samples in an experiment. Samples were mixed with a 2x loading buffer and boiled before loading into an SDS-PAGE gel or storing at −80°C for future use.

### Western blotting

Samples were resolved with 12% SDS-PAGE and transferred to a Whatman Protran nitrocellulose membrane using electrophoresis. Membranes were blocked for 30 min at room temperature with milk (5% milk in 1 × Tris Bufffered Saline with Tween20 (TBST)) and incubated overnight at 4°C with primary antibodies in milk. The following antibodies were used: αPan-CREB (1:5000), αPO_4_-CREB (1:1000), αLamin (1:5000), and αHSP70 (1:20,000). Following incubation in primary antibody, membranes were rinsed in TBST 4 × 15 min, blocked in milk, and incubated in secondary antibody (Jackson ImmunoResearch laboratories, Peroxidase AffiniPure Goat Anti-Rabbit IgG 111-035-003 or Goat Anti-Mouse 111-030-003) for 1 h at room temperature. After secondary antibody incubation, membranes were rinsed in TBST 4 × 15 min then incubated for 4 min in Pierce Western Blotting Substrate ECL reagent (Prod #32106). Membranes were exposed to film (Denville Scientific Premium Radiography Film, Cat #E3018) for time periods ranging from 5 s to 15 min prior to developing the film. Images of film were scanned and digitally saved using HP Scanner, version 2.4.4 (3).

During quantification, each dCREB2 band was normalized to the loading control (HSP70 for cytoplasmic samples, Lamin for nuclear samples). Values for each dCREB2 band are presented as fold change over the Zeitgeber Times (ZT0) value for that isoform. An appropriate film exposure length was selected for each isoform analyzed to ensure sufficient signal for the investigation.

### Statistical analysis

Behavioral data are presented as mean ± SEM. R (R Development Core Team, 2013) software was used for all statistical tests. The α-value was set to 0.05 for all assessments. For all behavioral experiments, independent two-sample *t*-tests were performed to provide between-subjects comparisons of animals trained or tested at different times during the day. To assess differences between individual and grouped time points, the Welch-Satterthwaite equation was employed to control for differences in sample size.

## Results

**Figure 1:**
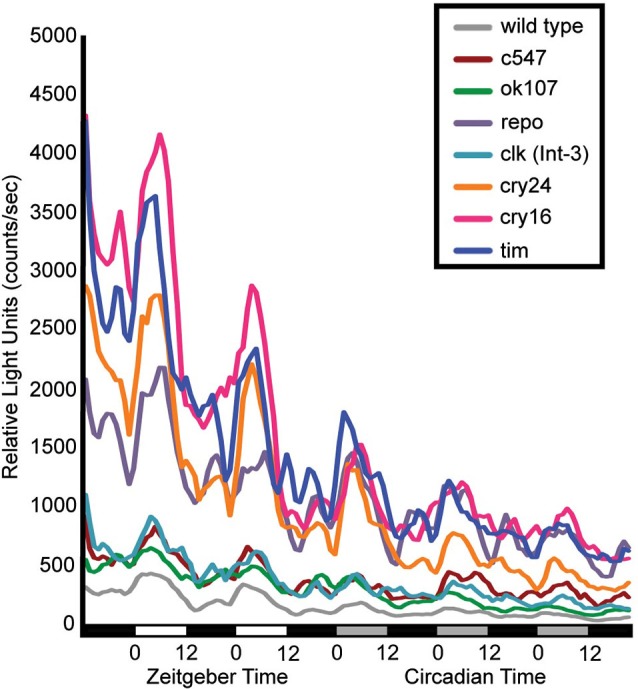
**dCREB2 transcriptional activity has a similar pattern of oscillation across cell populations**. Bioluminescence is plotted in multiple fly lines expressing a dCREB2 luciferase reporter under the control of tissue-specific GAL4 drivers. Each line represents the mean reporter activity over time (hours) across flies (*n* = 48) representing the progeny of UAS-FLP;CRE-F-luc transgenic flies crossed with wild type (gray), or GAL4 driver lines: *c547* (red), *ok107* (green), *repo*-GAL4 (purple), *clk(int-3)-GAL4* (teal), *crypGAL4-24* (cry24, orange) *crypGAL4-16* (cry16, pink), *tim*-GAL4 (blue). Each data point represents the average over three hourly time points. Light conditions are indicated along the x-axis first in LD (black: night, white: day), then in DD (black: subjective night, gray: subjective day).

Flies that contain a CRE-luciferase transgenic reporter show that dCREB2 transcriptional activity oscillates across the day/night cycle under the control of the circadian system (Belvin et al., 1999). When this report was published, it was not possible to determine whether this pattern of activity was characteristic of certain tissue and cell populations or was widespread. A second-generation reporter was made that allowed tissue-specific expression to be imposed on the reporter (Tanenhaus et al., 2012). When the reporter is expressed in different adult head tissues and cell clusters, the same oscillatory pattern is present in all areas where signal can be detected. Figure 1 shows the patterns of activity that are detected when reporter expression is restricted to different circadian and memory-related cell populations. Regardless of which brain cell types express the reporter, the same oscillating pattern of activity is evident. For this experiment, flies were entrained on a 12:12 light-dark schedule and CRE-luci activity in intact flies was measured for 2 days under light-dark conditions and 3 days under dark-dark conditions (the lighting conditions are indicated under the graph). As with the first generation reporter, CRE-luci activity oscillations persist into dark-dark conditions, indicating that these cell type-specific oscillations are also under circadian control. Diurnal peaks of activity are detected in sensory neurons of the visual system, integration neurons in the mushroom bodies, and in all of the different cell types of glia (Figure 1; Tanenhaus et al., 2012). These results lead to two predictions about dCREB2 activity. First, TOD may affect dCREB2 function in brain processes such as memory formation since most if not all brain regions show oscillations in activity. Second, since the cycling pattern is apparently ubiquitous, biochemical analysis of dCREB2 using an extract homogenized from all cells in the head could reveal part of the mechanistic bases for oscillatory activity.

**Figure 2:**
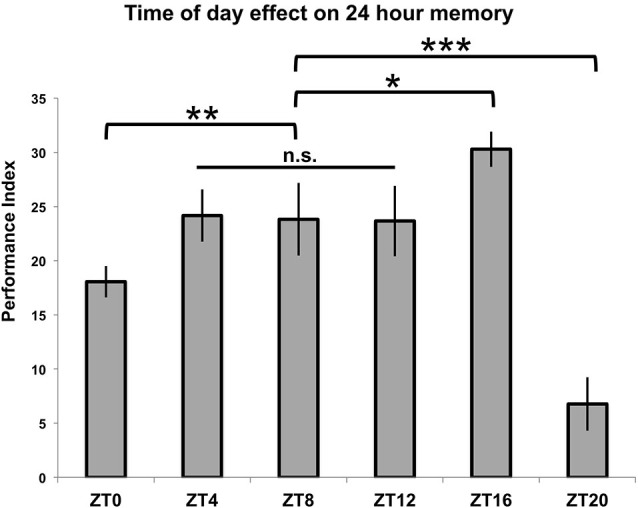
**Time of training affects 24-h memory**. Wild-type animals were entrained to a 12-12 light-dark schedule, trained with 7x spaced olfactory conditioning at six different Zeitgeber Times (ZTs) throughout the day, tested 24 h after training began (*n* = 7–8 for all groups). Performance index values are presented as experimental means and error bars indicate SEM. Animals trained at ZT4, ZT8, and ZT12 exhibited statistically similar memory scores (*p* > 0.9 for all individual comparisons between groups) and were therefore combined to serve as a baseline memory value for other comparisons. Animals showed diminished memory scores compared to baseline when trained at ZT0 (*p* < 0.01) and ZT20 (*p* < 0.001), while animals trained at ZT16 displayed enhanced memory retention (*p* < 0.05). Statistical differences between groups are indicated as * *p* < 0.05, ** *p* < 0.01, and *** *p* < 0.001.

In order to test TOD effects on memory formation, flies entrained to a 12:12 light:dark cycle were trained using seven cycles of repetitive spaced trials which began at six different time points (ZT = 0/24, 4, 8, 12, 16 and 20). These flies were then tested for memory formation 24 h later, at the time when their training began. The results are shown in Figure 2. For three of the time points (ZT = 4, 8 and 12), there is no difference in performance (*p* > 0.9 for all individual comparisons between groups), and these times are subsequently treated as a baseline level of performance. When training that starts at ZT = 16 is compared to this level, there is a significant enhancement of performance (*p* < 0.05). Conversely, there is a large, significant decrease in memory scores when training begins at ZT = 20 (*p* < 0.001) as well as a smaller decrease that is still significant when training begins at ZT = 0/24 (*p* < 0.01).

**Figure 3:**
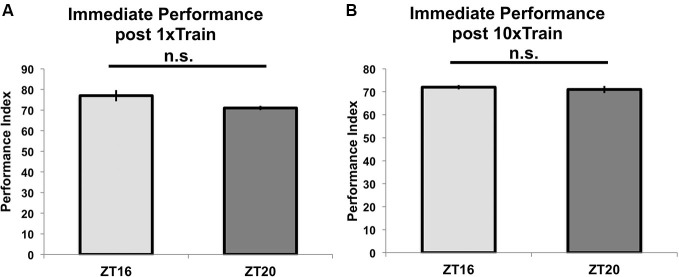
**Time of training does not affect learning or immediate memory**. Wild-type flies entrained to a 12-12 light-dark schedule were trained at either ZT16 or ZT20 with **(A)** a single trial of paired olfactory conditioning or **(B)** 10x spaced olfactory conditioning (*n* = 8 for all groups). Animals were tested immediately after training, and performance is presented as experimental means with error bars representing SEM. Flies trained with a single trial at ZT16 and ZT20 show statistically similar learning scores (*p* = 0.09). Likewise, flies given 10x spaced olfactory conditioning perform similarly (*p* = 0.8) when tested immediately after training.

To show that these differences in performance result from TOD effects on memory formation, we tested for TOD effects on learning at ZT = 16 and ZT = 20. Figure 3A shows that flies trained with a single training trial at ZT = 16 or ZT = 20 and tested immediately after the end of training perform similarly (*p* = 0.09), indicating that there is no TOD effect on learning under these experimental conditions. Correspondingly, flies trained with 10 (rather than 7) cycles of spaced training beginning at ZT = 16 or 20 and tested immediately after the end of training do not show any difference in performance (*p* = 0.8, Figure 3B). Since the immediate performance after 10 cycles of spaced training is identical whether the flies are trained at ZT = 16 or ZT = 20, it is unlikely that the major effect of the training time is through peripheral factors, such as olfactory acuity.

**Figure 4:**
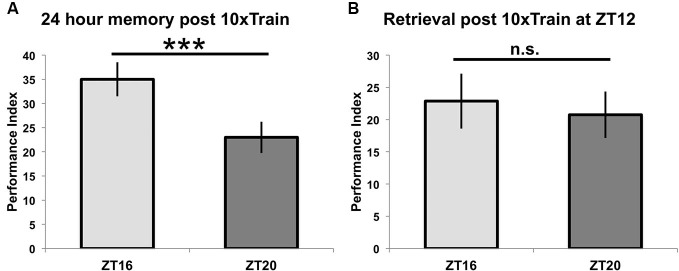
**Time of training but not time of testing affects long-term memory. (A)** Wild-type animals were entrained to a 12-12 light-dark schedule and trained at either ZT16 or ZT20 with 10x spaced olfactory conditioning, then tested 24 h later (*n* = 8 for both groups). Flies trained at ZT16 showed significantly better memory (*p* < 0.05) than flies trained and tested at ZT20, although the difference between groups is less than when flies were trained with 7x spaced conditioning (compare to Figure 2). **(B)** After entraining to a 12-12 light-dark schedule, wild-type flies were trained at ZT12 with 10x olfactory conditioning and tested for memory retention on the following day at ZT16 or ZT20 (*n* = 8 for both groups). Flies tested at different times showed statistically similar memory retention (*p* = 0.7). Data are presented as experimental means and error bars indicate s.e.m.

When different groups of flies are exposed to 10 cycles of spaced training beginning at ZT = 16 or ZT = 20 and tested for 24-h memory, there is a statistically significant difference between the two groups (*p* < 0.05), although the difference in memory scores is smaller than when flies are trained with seven spaced trials (*p* < 0.0001) (see Figure 4A, compare to Figure 2). In order to show that these effects on 24-h performance are not due to TOD effects on retrieval, flies trained at a common time (ZT = 12) and then tested for retrieval the next day at either ZT = 16 or ZT = 20 show identical performance (*p* = 0.7, Figure 4B). Since there are no differences in immediate performance between flies trained at ZT = 16 and ZT = 20 (Figure 3), and retrieval is identical (Figure 4), the differences in performance shown in Figure 2 are most likely attributable to TOD effects on the consolidation steps after immediate performance but before retrieval.

One important molecule that affects the formation of long-term memory is dCREB2. dCREB2 also affects the circadian clock, which presents a logical line of enquiry: does dCREB2 also contribute to the TOD effects on memory formation? We used Western analysis of whole head extracts to assess the status of dCREB2 protein isoforms across the day/night cycle. The dCREB2 gene utilizes alternative splicing and alternative translation initiation to make a large number of protein isoforms (Yin et al., 1995b; Tubon et al., 2013). These proteins in turn can be post-translationally modified in numerous ways, altering their mobility on denaturing protein gels and creating a large pool of dCREB2 isoforms which can presumably differentially affect dCREB2-mediated processes (Fropf et al., 2013).

For the sake of simplicity, we have historically grouped these different species into two functional categories, activators (A) and blockers (B). Since the activator functions in the nucleus, but most of the protein is sequestered in the cytoplasm, subcellular localization controls its function. Recently, we showed that the levels of a p35+ activator species transiently increase in the nuclear compartment after training that produces long-term memory (Fropf et al., 2013). This short-lived increase is most likely causally involved in the dCREB2-dependent events necessary for long-term memory formation. Because of these results, we examined the dCREB2 protein isoforms in the nuclear and cytoplasmic compartments across the daytime/nighttime.

Head extracts were made from flies entrained on a 12:12 light/dark cycle and collected at hourly time points. The extracts were separated into nuclear and cytoplasmic fractions and analyzed using Western blots. All 24 samples from across the day/night cycle were normalized for protein concentrations to ensure that equal amounts of protein were loaded in each lane. To confirm our equivalent protein load across lanes, the blots were cut along the 60 kD line and the upper portion of the blots were probed with a lamin- or Hsp70-specific antibody to verify roughly equal levels of nuclear or cytoplasmic protein (bottom image in each panel). Quantitating this experiment is technically very challenging, partly due to the number of samples (24) that have to be processed at one time, and partly because of the huge disparity in abundance between the normalization proteins (lamin and Hsp70) and the dCREB2 isoforms. In order to detect differences in the abundance of dCREB2 protein isoforms, large amounts of cytoplasmic or nuclear protein need to be analyzed.

**Figure 5:**
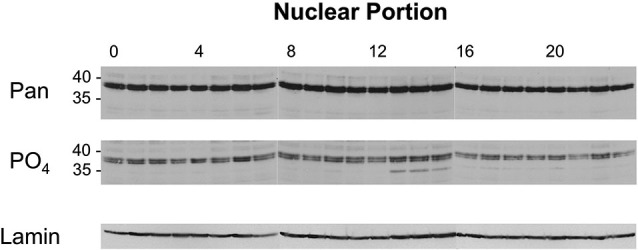
**Levels of nuclear dCREB2 protein isoforms oscillate through the day/night**. Representative Western blot images of nuclear dCREB2 isoform oscillation within a 24 h period. Wild-type animals were entrained to a 12-12 light-dark schedule and flies were collected at the beginning of each hour throughout the day. Heads were isolated and then separated into nuclear and cytoplasmic fractions for subsequent analysis using SDS-PAGE and Western blot. Each set of samples was probed with an α-Pan (top) and α-PO_4_ (middle) antibody to assess localization of dCREB2 isoforms across the day. Blots were probed with Lamin (bottom) as a loading control.

Figure 5 (top panel) shows the Western image for nuclear samples probed with two different dCREB2 antibodies. When the blots are probed with a pan-dCREB2 antibody, the major identifiable bands are the doublet blocker species that run with an apparent molecular weight near 40 kD (top panel). There is no readily apparent large difference in the amounts of these proteins across the day/night cycle, a result that is consistent with previous data (which only sampled 6 time points) using a dCREB2-specific monoclonal antibody. However, when the blots are probed with the S231 phospho-specific antibody, there is a clear increase in the p35+ species between ZT = 13 and ZT = 15. Although a longer exposure shows that this species can be detected in nuclear samples from all time points, its levels increase when nuclear extracts are sampled during this time window.

**Figure 6:**
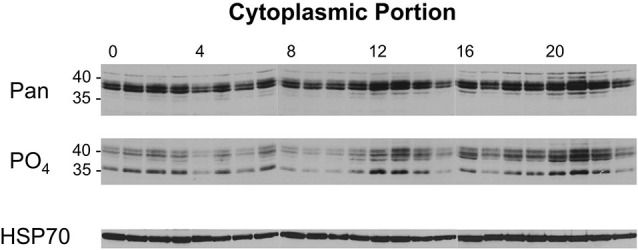
**Levels of cytoplasmic dCREB2 protein isoforms oscillate through the day/night**. Representative Western blot images of cytoplasmic dCREB2 isoforms show oscillation within a 24-h period. After entraining to a 12-12 light-dark schedule, wild-type animals were collected at the beginning of each hour throughout the day, heads were separated, and cytoplasmic protein was isolated for each sample. Samples were analyzed using SDS-PAGE and Western blot and were probed with HSP-70 as a loading control. To analyze dCREB2 isoform levels across the day, cytoplasmic samples were probed with an α-Pan (top) and α-PO_4_ (middle) antibody.

A similar analysis was performed with the cytoplasmic samples from flies collected across the day/night cycle, and these results are presented in Figure 6. When these samples were probed with the pan-dCREB2 antibody, three changes occurred across the day/night cycle. First, there appears to be a slight decrease in the amount of blocker protein around ZT = 4–6. Additionally, there is a noticeable increase during the ZT = 12–14 period, and an even larger increase during the ZT=20–22 period. These changes are corroborated with the S231 phospho-specific antibody; although there are minor differences between the two antibodies, the general expression pattern is very similar.

**Figure 7:**
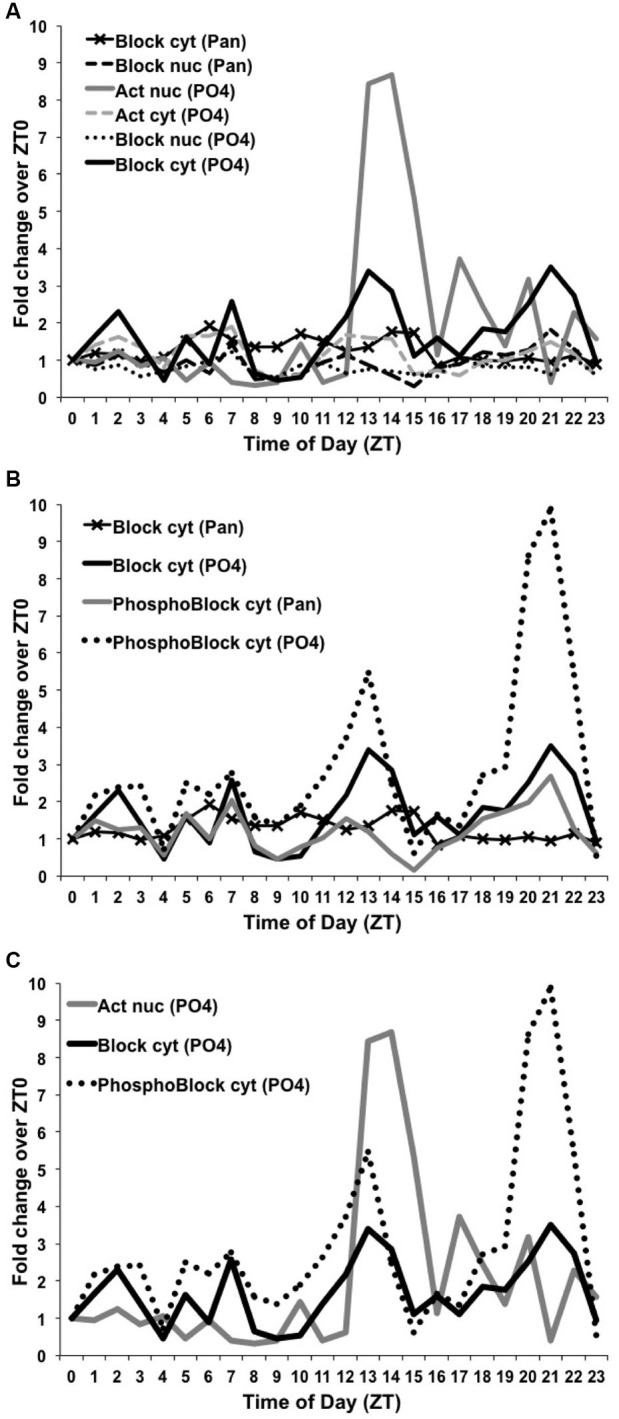
**Levels of dCREB2 protein isoforms oscillate through the day/night**. Quantification of Western blot images from samples generated by wild-type flies collected each hour across a day (see Figures 5 and 6). Each dCREB2 band was normalized to the appropriate loading control across time points. Data are presented as fold changes of the normalized values for each isoform compared to the normalized ZT0 value for that isoform. p35 Activator, p40 Blocker, and hyperphosphorylated p40+ Blocker dCREB2 species (labeled “Act”, “Block”, and “PhosphoBlock”, respectively) are presented for both nuclear (nuc) and cytoplasmic (cyt) samples. **(A)** Illustrates the nuclear increase in Activator during the early night (solid gray line) and fluctuating levels of cytoplasmic Blocker, which show multiple peaks and troughs (solid black line). **(B)** Highlights the strong early- and late-night peaks in cytoplasmic, hyperphosphorylated Blocker species (dotted black line). **(C)** Three dCREB2 species have time points with greater than 2-fold increases over ZT0 values.

To quantify these differences, we used ImageJ to measure the dCREB2-specific signal intensity for both dCREB2 antibodies and the corresponding loading control for each of the 24 time points. The dCREB2 signal at each time point was then normalized to its loading control, and the value at ZT = 0 is set to 1. All other time points across the daytime and nighttime are expressed relative to the ZT = 0 value. Figure 7 shows the relative fold change for each time point plotted as a function of the zeitgeber time (ZT). Panel 7A shows the two plots for the activator species (nuclear and cytoplasmic) detected with the α-PO_4_ antibody (based on the data shown in Figure 5), and all four plots (based on the data presented in Figure 6) for the blocker (either nuclear or cytoplasmic compartments probed with either the α-Pan or α-PO_4_ antibody). For the activator, the most noticeable change is the abundance of the nuclear activator species (shown in solid gray in Figure 7A) during the ZT = 12 to ZT = 16 window. There is almost a nine-fold increase in its abundance during this period, with smaller peaks later on during the nighttime.

To the naked eye, there seems to be a late nighttime increase in the doublet that corresponds to blocker-related species (Fropf et al., 2013), but there is also a clear increase in a cluster of bands (see Figure 6) that are most likely to be hyperphosphorylated forms of the blocker (Zhang et al., 2013). The molecular identity of each band is unclear, since there are at least eight phosphorylation sites that are present on the dCREB2 blocker proteins, and which are conserved on all CREB family members. It is likely that combinatorial/differential phosphorylation contributes to these small differences in apparent mobility. Regardless of their molecular distinctions, this cluster of bands increases during the late nighttime, and this increase is detectable with both antibodies. Figure 7B shows the different quantitative patterns that are seen with both antibodies for the hyperphosphorylated and unphosphorylated or mildly phosphorylated (doublet) blocker species. The largest increase is seen with the hyperphosphorylated blocker species (dotted black trace in Figure 7B) detected using the αPO_4_ antibody, but a qualitatively similar pattern is detectable using the α-Pan antibody (solid black trace). Consistent with these results, the doublet bands detected using the α-PO_4_ antibody (dark gray in Figure 7B) also show a late nighttime peak. Finally, there is a general decrease in blocker and hyperphospho-blocker expression during the mid-day period, although this general effect is not as striking as the nighttime changes in abundance.

Figure 7C summarizes the dynamic changes in abundance that we believe to be most functionally important. This plot illustrates the large increase in nuclear activator (dark gray trace in Figure 7C) during the early evening. It also highlights the increases in hyperphospho-blocker (dotted black trace) and blocker (solid black trace) during the late nighttime.

## Discussion

Our analysis shows that dCREB2 activity oscillates (probably in all tissues) across the daytime and nighttime, and that this pattern of activity is under circadian control. In this report, we show that the TOD affects long-lasting memory formation, with a peak in performance during the early nighttime and a significant trough in the late night. Our experiments do not rigorously show that the TOD effect is under circadian control, nor that the TOD is affecting bona fide long-term memory. However, since dCREB2 activity oscillates under circadian control, and its protein isoforms show patterns of abundance that change across the day/night cycle, the simplest explanation is that the circadian-controlled oscillations in dCREB2 activity are partly responsible for the TOD effects on behavior. This would implicate TOD affecting LTM, since dCREB2 is not required for ARM, but further experiments are needed to clarify this point. Our biochemical analysis is the first report that levels of multiple dCREB2 proteins oscillate across the daytime and nighttime. These observations raise several intriguing possibilities about the molecular mechanisms underlying TOD effects on memory formation.

Previous work in invertebrate systems, including the fly, shows that olfactory processing is under circadian control, with a distinct peak in acuity that occurs during the early nighttime (Krishnan et al., 1999, 2001; Decker et al., 2007). Our behavioral experiments show a TOD memory peak during the early nighttime as well as memory troughs late at night and at the night/day transition. Is it possible that our memory effects are attributable to TOD effects on olfactory acuity? Although formally possible, we think this is unlikely. Neither single cycle training, nor repetitive spaced training, produces differences in immediate performance tested at the end of training. In neither case are the performance scores “saturated”, indicating that subtle effects of olfactory acuity would most likely be measurable. In the case of repetitive spaced training, the TOD also does not affect retrieval. Therefore, it is most likely that the observed differences in performance are due to differences in molecular and cellular processes that occur after training but prior to retrieval (ie., during consolidation).

The Western analyses of dCREB2 protein isoforms suggest part of the mechanistic underpinnings of the oscillating CRE-luciferase pattern and the TOD behavioral results. Using the simple categorization of protein isoforms into activators or blockers, the heuristic activator-to-blocker ratio (A/B) is a useful preliminary means by which to correlate activity and behavior with molecular data.

The timing of the nighttime peak in CRE-luciferase activity could be attributable to the increase in nuclear levels of p35+ and the resulting increase in the A/B ratio. During the falling phase of the nighttime peak (ZT = 17–20) in activity, there is a noticeable increase in blocker-related species, which is predicted to decrease the A/B ratio. These dynamic fluctuations in protein level closely correspond to the changes in CRE-luciferase reporter activity.

Our results demonstrate notable correlations between the TOD behavioral effects and the circadian profile of dCREB2 proteins. At ZT = 20, there is a significant depression in memory formation, an event which coincides with apparent increases in blocker-related species clearly visible on the Western blots. At ZT = 16, we measured a significant increase in performance. This time point correlates with the end of a window (ZT = 13–15) when nuclear levels of the activator are elevated. Based on these relationships, we hypothesize that the dynamics of dCREB2 protein levels contribute to the TOD effects on memory formation. A simple prediction based on these data is that optimal behavioral enhancement might occur if training begins between ZT = 13 and ZT = 15.

dCREB2 has a complex set of post-translational modifications that occur on the various protein isoforms. Our classification (activator vs. blocker) and the formulation of an A/B ratio are undoubtedly very simplistic. For example, a number of different protein isoforms probably can contribute to terms in the numerator and denominator. Similarly, the various post-translational modifications that can occur likely contribute to a specific protein’s function as either an activator or a blocker. Despite this oversimplification, the use of the A/B ratio as an analytical tool produces a good correlation between the protein fluxes and the temporal peaks and troughs in activity and memory formation.

The A/B ratio can change due to increases or decreases in the numerator or denominator. It is interesting that at least two out of the four possibilities seem to occur at different points in time, suggesting that the accompanying molecular mechanisms are likely to be distinct. During the early nighttime, the A/B ratio is changed since more nuclear activator is detected, and this probably contributes to the nighttime peak in CRE-luciferase activity, and the TOD peak in memory formation. Conversely, during the late nighttime, the A/B ratio decreases because the different blocker species increase. We believe that there is a stoichiometric excess of blocker isoforms in the cytoplasmic compartment and that the majority of this protein exists as homodimeric species (B:B). Through unknown mechanisms, the blocker protein seems to be tethered in the cytoplasm. The activator proteins are much less abundant than the blockers, and this disparity might result in the majority of the activator protein existing in A:B heterodimers, which are consequently tethered in the cytoplasm. If the overall levels of the blocker proteins decrease, then the levels of A:A homodimers might increase and be able to enter the nucleus. Alternatively, A:B heterodimers might respond to signaling events and dissociate, allowing activator monomers or A:A homodimers to form and enter the nucleus. The apparent increase in nuclear p35+ during the early nighttime is most likely due to an increase in nuclear entry as described previously during memory formation (Fropf et al., 2013). However, it is formally possible that it is attributable to an increase in synthesis of the activator, or a decrease in nuclear export or degradation of the activator. Future experiments are needed to distinguish between these possibilities.

Although it is impossible at this point to distinguish amongst all of these possibilities, the important conclusion is that different underlying mechanisms seem to be employed across the day/night cycle to alter transcriptional activity. Future work will determine if the known circadian signaling pathways (cAMP, MAPK, NO, casein kinases) are employed at different times to contribute to these changes. Both the MAPK and cAMP/PKA/CREB pathways have emerged as crucial components in circadian biology and memory formation. Additionally, molecular components from both pathways show oscillatory patterns in their abundance or activity across the day/night, and these peaks and troughs correlate with, or are causally involved in, memory formation. Taken together, it is highly plausible that these and other molecules and pathways also contribute to the ebb and flow of memory formation across the day/night cycle.

*Drosophila* employs elaborate anatomical circuitry to process and maintain long-term memory for olfactory conditioning (Davis, 2011; Pitman et al., 2011; Wu et al., 2011; Chen et al., 2012; Pai et al., 2013). In addition to the central complex, various subdivisions of the mushroom bodies, and associated pairs of neurons such as DPM/APL/DAL, other regions or clusters of neurons are undoubtedly involved. The participation of a particular group of neurons is also likely to begin at the time of training and change during the duration of the consolidation period, which probably lasts for a few days after the end of training. This dynamic situation, in which different neurons are communicating with each other and synaptic plasticity is used to strengthen/weaken transient connectivity, probably includes both increases and decreases in dCREB2-mediated activity in different cell populations. Given the complexity of both the anatomical and molecular substrates for memory formation, it is remarkable that merely altering the initial time of training can have significant effects on such an intricate process. Although simplistic, we think the primary effects of training time on memory formation occur during or soon after the time of training, as opposed to effects during consolidation and retrieval. Since the current data with the second generation CRE-luciferase reporter suggest that most, if not all, cells in the fly head have synchronous, oscillatory dCREB2 activity, we believe that these activities, rather than ones that we cannot detect using our reporter, are responsible for the behavioral TOD effects. However, further experimentation would be needed to confirm this point.

While it may hold intuitive appeal that the worst window for memory formation occurs during the late nighttime, when flies are typically asleep, it is not as clear why the best period would also be during the night. One possibility is that other systems-level processes occur during the early nighttime sleeping period, and these processes use some of the same molecules and signaling pathways that are used for memory formation. In mammals, there is strong evidence that the different types of patterned neuronal activity (e.g., slow wave sleep, rapid eye movement sleep) contribute to memory consolidation (Walker and Stickgold, 2004; Marshall and Born, 2007). Of particular interest is the recent identification of cAMP and phospho-CREB peaks during REM sleep in mice (Luo et al., 2013). Whether fly sleep shows different electrophysiological or behavioral states is an active area of research, but there is nothing that currently distinguishes the early-from-late nighttime sleeping state (van Swinderen et al., 2004; van Alphen et al., 2013). However, it is interesting that during the late night flies sleep less and exhibit greater fragmentation of their sleep. Further technical advances are needed to explore these intriguing ideas.

Another possibility is that the peaks and troughs in memory formation could be related to circadian processes that normally occur during the nighttime and are not limited to anatomical regions that process light. Our behavioral analysis was done under light:dark conditions (rather than in constant darkness), thus precluding a strict circadian interpretation of our results. Nonetheless, the simplest hypothesis is that circadian influences affected the TOD behavioral experiments. In mammals, phase delays during the early nighttime are known to involve the CREB pathway and result in the phosphorylation and activation of CREB without an accompanying change in the levels of unphosphorylated CREB protein (Ginty et al., 1993; Ding et al., 1997). During the late nighttime, light triggers phase advances, and these alterations in phase ultimately affect the phosphorylation state of CREB. However, the signaling pathways between light reception and CREB probably differ between the early and late night, and the resulting transcriptional effects are certainly different, since they have opposite effects on the phase of the clock. Behavioral training during the early nighttime may utilize specific signaling pathways that are normally peaking during this time, resulting in performance enhancement. Conversely, the general signaling state during the late nighttime may interfere with dCREB2 activation that is normally required for memory formation, thus resulting in poor behavioral performance when flies are trained at this time. Mechanistically linking circadian signaling with dCREB2 activity will require more knowledge about the signaling pathways used for phase delays and advances as well as the molecular events that determine the synthesis, degradation and nuclear transport of dCREB2 proteins. Regardless of the definite molecular details, our data clearly demonstrate a strong correlation between the activity of dCREB2, its role in a major neuronal function (memory formation), and its protein levels across the 24-h circadian cycle.

## Conflict of interest statement

The authors declare that the research was conducted in the absence of any commercial or financial relationships that could be construed as a potential conflict of interest.
